# Vitamin D Status in Epileptic Children on Valproic Acid; a Case-Control Study

**Published:** 2020-02-27

**Authors:** Ameena Taha Abdullah, Zaher Taher Mousheer

**Affiliations:** 1 Raparin Pediatric Hospital, Hawler Medical University, College of Medicine, Erbil, Iraq.

**Keywords:** Epilepsies, myoclonic, valproic acid, cholecalciferol (vitamin D3)

## Abstract

**Introduction:**

Much attention has been paid to the association between valproic acid treatment and bone health. The objective of this study is to compare the serum vitamin D3 level in the epileptic children under valproic acid treatment with the healthy control group.

**Methods:**

A case-control study has been carried out to compare vitamin D3 levels in 50 epileptic children who were treated with valproic acid with 50 healthy children selected from children visiting the hospital for routine checkup as control group.

**Results:**

100 cases with the mean age of 7.57± 3.62 years (range: 2 – 15 years) were studied (44% boys). Among the 50 epileptic cases; 41 (82%) had generalized and 9 (18%) had partial seizure (56% well controlled and 44% poorly controlled). 15 (30%) of epileptic cases were using anti-epileptic drugs for 6-12 months, 36% for 12-24 months, and 34% for more than 24 months. The case and control groups were similar regarding gender (p =0.99), age (p = 0.24), and BMI (p = 0.64). 49 (49%) patients had some grade of vitamin D3 deficiency. There was a significant difference between case and control groups regarding vitamin D3 levels (p = 0.001). None of the controls had severe vitamin D3 deficiency, while 14% of cases did. 36 (72%) individuals in control group had sufficient or optimal vitamin D3 levels; while only 15 (30%) case patients had such levels. Generally, the control group had higher vitamin D3 levels in comparison to case group (p = 0.001).

**Conclusions:**

The study revealed that there was a higher prevalence of vitamin D3 insufficiency in epileptic children receiving valproate monotherapy compared with healthy children. Vitamin D3 supplementation should be given to all epileptic children even before initiation of anti-epileptic drugs.

## Introduction

Epilepsy is defined as a condition of susceptibility to recurrent seizures, when at least two or more unprovoked seizures occur more than 24 hours apart (1). 

The clinical diagnosis of epilepsy usually requires the occurrence of at least one unprovoked epileptic seizure with either a second seizure or enough electroencephalogram (EEG) and clinical information to convincingly demonstrate an enduring predisposition to develop recurrences. Antiepileptic drugs are used for prevention of seizure; carbamazepine, sodium valproate, phenytoin and phenobarbitone are commonly used antiepileptic drugs (2). The effects of antiepileptic drugs on vitamin D3 levels have been studied for more than 40 years (3, 4). Most of the antiepileptic drugs are inducers of hepatic CYP450 metabolism. These antiepileptic drugs result in increased hepatic metabolism of vitamin D leading to low vitamin D3 levels. However, some non-enzyme inducing antiepileptic drugs (e.g. valproic acid) have also been associated with low vitamin levels, which cause poor bone health (5). 

Much attention has been paid to the association between valproic acid treatment and bone health over the last few years. A recent study revealed that treatment with valproic acid could lead to a decrease in vitamin D3 levels in pediatric epileptic patients, which explained the adverse bone-related side effects of valproic acid therapy (6). Vitamin D is an important prohormone, which plays roles in metabolism of calcium, strengthening the bones and other metabolic processes (7, 8). 

Approximately 1% of the population is on long-term and sometimes lifelong antiepileptic drug therapy and therefore, exposed to the potential metabolic side-effects of these drugs. These adverse effects are changes in homocysteine, lipoproteins and vitamin D metabolism (9). Multiple health outcomes are dependent on an adequate vitamin D3 level. Vitamin D is very important for proper growth and development of bones in children. Type, dosage and duration of antiepileptic drugs determine the extent of osteopathy (10). Vitamin D3 deficiency is a worldwide condition (11). About one billion people in the world have vitamin D3 insufficiency or deficiency (<30 ng/mL) (12). The most important cause of vitamin D3 deficiency is long-term use of antiepileptic drugs in epileptic children (13, 14). Since they need long-term anticonvulsant therapy, they have a high risk for showing side effects (15). Long-term use of anticonvulsant medications has been associated with high incidence of rickets, higher risk of fracture, and decreased bone mineral density (16, 17).

Vitamin D3 supplementation is associated with decreased frequency of seizures, because it regulates proconvulsant and anticonvulsant factors; also vitamin D is involved in the down regulation of cytokine IL-6, which is a proconvulsant (18). The objective of the study is to compare the serum vitamin D3 levels of the epileptic group, who were treated with valproic acid, with the healthy group as control. 

## Methods

***Study design and setting***

This retrospective case-control study has been carried out to determine the serum level of vitamin D3 in 50 epileptic children who were treated with a single antiepileptic drug (valproic acid) and compare it with the control group comprised of 50 children with normal growth parameters selected from those attending the pediatric clinic at Raparin Pediatric Hospital in Erbil city, Kurdistan region, Iraq, from march 1st, 2019 to September 1st, 2019 (six month).

 The protocol of the study was approved by the Research Ethics Committee of Kurdistan Board for Medical specialties (Ethics code: IRAQ.KBMS.2019.177). Informed Consent (oral and written) was taken from all child parents.

***Participants***

All the children had normal age-appropriate development. The case group included epileptic children 2-15 years old under regular treatment with valproic acid for 6 months or more. Children with neurological deficits like cerebral palsy or mental retardation, children with episodes of febrile convulsion, epileptic children who are seizure free and have stopped taking medication for three or more years, children with chronic diseases like renal, hepatic, endocrine or metabolic bone diseases, and children with vitamin D3 supplementation were excluded from the study.

***Data gathering***

A questionnaire was designed for the study including: identity; name, date of birth, sex, and body mass index (BMI), which was calculated by weight in kilograms divided by the height in square meters (18). According to Centers for Disease Control and Prevention (CDC) chart, the underweight is less than the 5th percentile; normal weight between 5^th^-85^th^ percentile and overweight is more than 85^th^ percentile. History of epilepsy; type and frequency of seizures over the last 3 months, to define well or poorly controlled cases, (well controlled case was defined as seizures free for the last 3 months) (1), and duration of the antiepileptic drugs was taken (20). 

***Vitamin D3 level measurement***

Two milliliters of peripheral venous blood was drawn from the child. Samples were sent to laboratory of Raparin teaching Hospital and analysis was done for 25-Hydroxy vitamin D level via ELISA test. Severe vitamin D3 deficiency was defined as a level less than <5 ng/ml; deficiency was defined as the level between (5-15 ng/ml); insufficiency was defined as the level between (15-20 ng/ml); and between (20-30 ng/ml) vitamin D was deemed sufficient. Optimal level was defined as the level between (30-50 ng/ml) and upper normal was defined as the level between (50-70 ng/ml). 

***Statistical analysis***

The statistical package for the social sciences program version 24 (SPSS, IBM Company, Chicago, IL, USA), was used for data analysis. Findings were reported as mean ± standard deviation or frequency (%). The results were analyzed using frequency distribution and t-test and Chi square or Fisher’s Exact tests if necessary. P-value of ≤ 0.05 was considered as statistically significant. Sample size was calculated using Epi Info™ version 7.

## Results

***Baseline characteristics of cases***

100 cases with the mean age of 7.57± 3.62 (range: 2 – 15) years were studied (44% boys). Table 1 shows the baseline characteristics of studied cases. Among the 50 epileptic cases 41 (82%) had generalized and 9 (18%) had partial seizure (56% of the seizures were well controlled and 44% poorly controlled). 15 (30%) of epileptic cases were using anti-epileptic drugs for 6-12 months, 36% for 12-24 months, and 34% for more than 24 months. Table 2 compares the baseline characteristics as well as vitamin D3 levels between case and control groups. The case and control groups were similar regarding gender (p =0.99), age (p = 0.24), and BMI (p = 0.64). 

***Vitamin D3 levels***

49 (49%) patients had some grade of vitamin D3 deficiency. There was a significant difference between case and control group regarding vitamin D3 levels (p = 0.001). None of the controls had severe vitamin D3 deficiency, while 14% of cases did. 36 (72%) controls had sufficient or optimal vitamin D3 levels; while only 15 (30%) cases had such levels. Generally, the controls had higher vitamin D3 levels in comparison to cases (p = 0.001).

## Discussion

In this study, we observed that the level of vitamin D3 was significantly lower among epileptic children on valproic acid monotherapy compared to healthy children, a similar result was concluded by Khadum et al. and Rafiq et al. (21, 22). 

In our study, we revealed that more than two third of patients had sub optimal vitamin D3 level, while less than one third of them had optimal vitamin D3 level; this result is in agreement with Menon et al. study (23).

There was no significant statistical association between study group (case or control) and age or BMI of children with decreased level of vitamin D3. This is in agreement with Alison et al. study (24) and in contrast to a previous study done by Baek et al. (25), which showed that the level of vitamin D3 decreases with increase in BMI (children with high BMI have a high body fat, which acts as a reservoir for lipid-soluble vitamin D). Our study showed that epileptic girls had lower vitamin D3 levels than boys. Other studies of the population, which included healthy children also showed lower levels of vitamin D3 and a higher frequency of vitamin D3 deficiency in girls compared to boys (11). Unfortunately, our study does not explain the reason for this finding but it could be due to the difference in the amount of sun exposure as the duration of outdoor activities is shorter in girls compared to boys. The current study revealed that there is a significant association between the non-enzyme inducing antiepileptic drug sodium valproate and vitamin D3 level; this can be explained through the enzyme inhibiting effect of valproic acid, which affects vitamin D3 metabolism. This result is in agreement with Xu et al. study (6) .

Routine monitoring of vitamin D level is warranted to prevent vitamin D3 deficiency in epileptic children on chronic valproate therapy. Vitamin D3 supplementation should be given to all epileptic children, even before initiation of antiepileptic drugs, and these patients should follow a well-balanced diet and healthy lifestyle to optimize seizure control.

**Table 1:** Baseline characteristics of studied cases

**Variables**	**Number (%)**
**Gender**	
Girl	56 (56.0)
Boy	44 (44.0)
**Age (year)**	
2-5	33 (33.0)
5-10	38 (38.0)
10-15	29 (29.0)
**Body mass index**	
< 50	41 (41)
50-85	38 (38)
>85	21 (21)
**Vitamin D level**	
Severe deficiency	7 (7.0)
Deficiency	21 (21.0)
Insufficiency	21 (21.0)
Sufficient	28 (28.0)
Optimal	23 (23.0)

**Table 2:** Comparing the baseline characteristics as well as vitamin D level between case and control groups

**Variables**	**Study groups**		**p-value**
	**Case (n = 50)**	**Control (n = 50)**	
**Gender**			
Girl	28 (56)	28 (56)	0.99
Boy	22 (44)	22 (44)	
**Age (year)**			
2-5	15 (30.0)	18 (36.0)	0.24
5-10	23 (46.0)	15 (30.0)	
10-15	12 (24.0)	17 (34.0)	
**BMI**			
< 50	21 (42.0)	20 (40.0)	0.64
50-85	17 (34.0)	21 (42.0)	
>85	12 (24.0)	9 (18.0)	
**Vitamin D level**			
Severe deficiency	7 (14.0)	0 (0.0)	0.001
Deficiency	14 (28.0)	7 (14.0)	
Insufficiency	14 (28.0)	7 (14.0)	
Sufficient	6 (12.0)	22 (44.0)	
Optimal	9 (18.0)	14 (28.0)	

**BMI: **Body mass index.

## Limitation

Our study had some limitations, the main one being that vitamin D3 levels were not measured before starting valproic acid therapy and hence, we cannot categorically attribute the levels to antiepileptic drug use. Second, we did not assess bone density in both cases and controls due to both financial reasons and lack of a standardized reference range for children. Third, sample size was small. Fourth, we did not study the participants’ lifestyle, like diet, activity and sunlight exposure, which might have affected vitamin D3 level. Strengths of our study were that we recruited cases and controls from the same hospital; thus, reducing difference based on ethnicity, social customs and socioeconomic status. 

Additionally, the present study has a retrospective design, which has limitations in its nature such as missing data. Short duration of study, not following the patients, and being a single centered study were among other limitations of the study. 

## Conclusion

The study revealed that there is a higher prevalence of vitamin D3 insufficiency in epileptic children under valproate monotherapy compared with healthy children. Further studies are warranted to determine other important factors that contribute to low vitamin D3 levels in children on valproate therapy. 
